# Coming down the “chimney”: Percutaneous coronary intervention on a post‐transcatheter aortic valve replacement with valve‐in‐valve patient

**DOI:** 10.1002/ccr3.6435

**Published:** 2022-10-08

**Authors:** Adam Taleb

**Affiliations:** ^1^ Athens Medical Center Athens Greece

**Keywords:** aortic valve, cardiovascular disease, percutaneous coronary intervention, valve replacement

## Abstract

Accessing the coronary arteries post‐transcatheter aortic valve replacement is a growing challenge. This is a case of a patient requiring percutaneous coronary intervention on a left circumflex artery after a TAVR valve‐in‐valve and a left main “chimney” stent, describing all the challenges met during the procedure.

## Graphical abstract

Accessing the coronary arteries post transcatheter aortic valve replacement is a growing challenge. This is a case of a patient requiring percutaneous coronary intervention on a left circumflex artery after a TAVR valve‐in‐valve and a left main “chimney” stent to prevent coronary occlusion were performed.

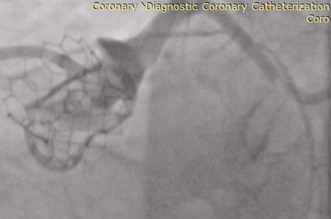

## 1 INTRODUCTION

The use of transcatheter aortic valve replacement (TAVR) is expanding, as recent trials showed favorable outcomes in intermediate and low surgical risk patients. This is leading to an increased number of TAVR procedures, going along with the spirit of the time for less invasive techniques. One of the concerns about TAVR is the ability to access the coronary arteries after the procedure,^1^ which becomes even more critical when TAVR is performed on low‐risk patients. These patients are in general young, with less comorbidities, making the risk of developing critical coronary artery stenosis years after the TAVR a real concern. The complexity increases when TAVR is performed on a patient who already had surgical replacement of the aortic valve in the past, so a valve‐in‐valve procedure was performed.

To raise the bar of complexity, in patients with low takeoff of the left main coronary artery and high risk of coronary artery occlusion, a stent is deployed in the ostium of the left main, protruding outside the perimeter of the TAVR valve into the aorta like a “chimney,” to ensure patency and prevent a catastrophic event.^2^ This is called “chimney” stenting technique, where the stent extends from the proximal part of the endangered coronary artery, in parallel to the TAVR valve and cranially, creating a lumen for perfusion between the displaced leaflets and the aortic wall.^3^

## 2 CASE

This is a case of a 76 years old female patient, with past medical history of severe aortic stenosis, who initially underwent surgical aortic valve replacement with a bioprosthetic valve, which got degenerated and patient developed dyspnea with exertion. The surgical valve was stenotic, so a valve‐in‐valve TAVR with balloon‐expandable valve was performed. Due to low takeoff of the left main coronary artery, a drug‐eluting stent was deployed right after the TAVR valve deployment with the “chimney technique” in the ostium of the left main coronary artery. Two years after the TAVR procedure, she developed progressive angina with even mild exertion.

## 3 DIFFERENTIAL DIAGNOSIS

The differential diagnosis for this patient included but was not limited to prosthetic valve degeneration (stenosis or regurgitation), coronary artery disease (causing angina), or new native valvular disease (causing elevated pulmonary artery pressures).

## 4 INVESTIGATIONS AND MANAGEMENT

**FIGURE 1:**
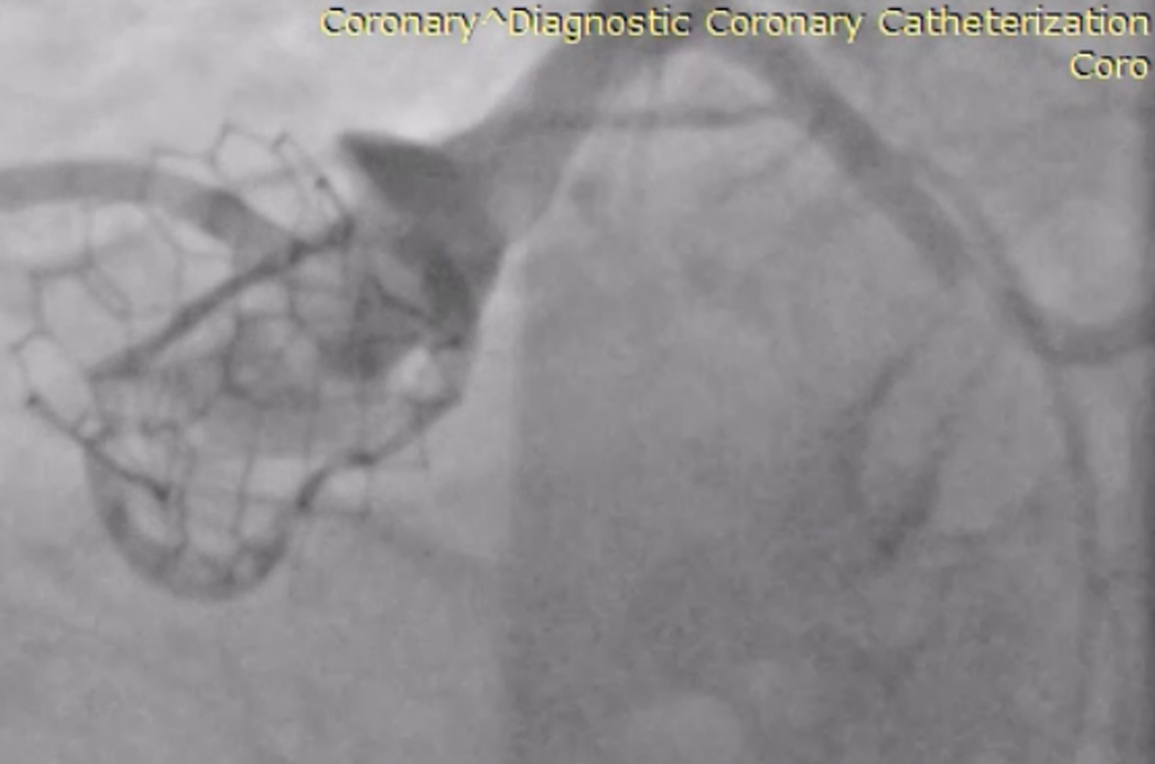
Lesion on the middle segment of the left circumflex artery prior to the percutaneous intervention

Initial work up included a transthoracic echocardiogram that showed preserved left ventricular ejection fraction of 55%, a mean gradient of 16 mmHg across the aortic valve prosthesis, normal wall motion, and normal native valves function. A myocardial perfusion study was performed, which showed ischemia on the lateral and anterolateral wall of the left ventricle. A coronary angiogram was performed that showed 95% stenosis on the middle segment of the left circumflex artery, consistent with the findings of the myocardial perfusion scan (Figure 1).

The challenges of treating a lesion with this anatomy include engagement of the oval‐shaped left main chimney stent, behind the TAVR and the surgical valve, passing an angioplasty wire through the deformed chimney stent, crossing the lesion, and delivering a stent through all the hardware in the left main ostium. Of major concern is crossing the left main chimney stent through the lumen and not from a side strut, as this would increase the risk of getting the new stent trapped in the struts of the left main stent and not being able to push in or pull back, thus compromising the flow into the coronary system and possibly making the patient hemodynamically unstable.

**FIGURE 2:**
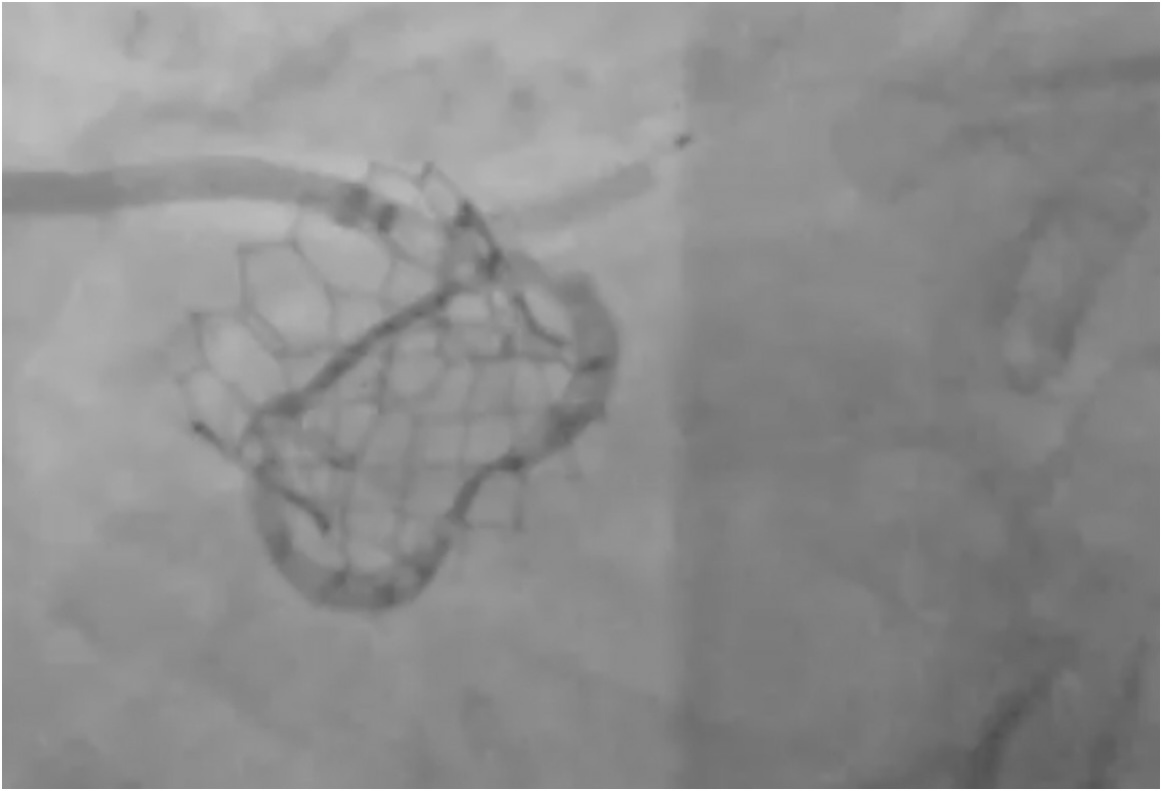
Balloon inflation to assist guide extension delivery through the left main “chimney” stent

**FIGURE 3:**
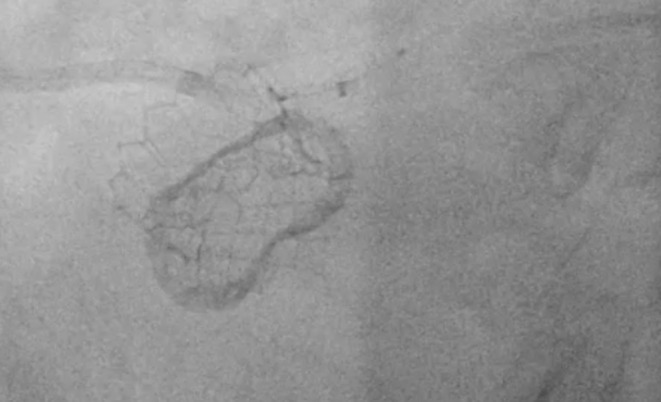
Balloon assisted guide extension delivery through the left main “chimney” stent

**FIGURE 4:**
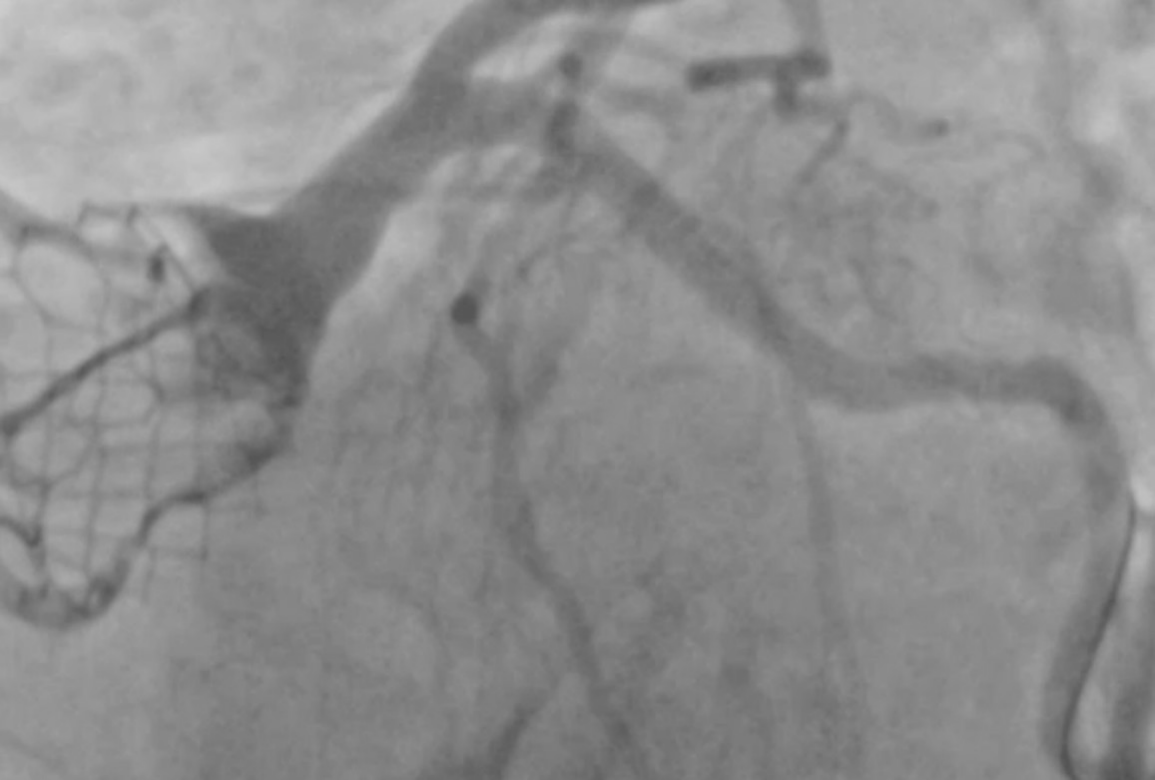
Middle segment of the left circumflex artery post‐percutaneous intervention

A 7 Fr AL 1.5 guide catheter was used to engage the chimney stent. An 0.014 inch guidewire with a manually formed J‐shaped tip was used to cross the deformed stent. The purpose of the J‐shaped tip was to create a loop and pass through the lumen of the stent, minimizing the risk of getting out of a side strut and re‐entering more distal. After the wire reached the left anterior descending artery, an uninflated balloon was advanced to assess the accurate placement of the wire through the lumen of the existing stent. There was no resistance noted pushing the balloon over the wire. With the support of the balloon and the wire, an attempt was performed to deliver a 6 Fr guide extension catheter in the distal segment of the left main coronary artery. It was not successful, as there was resistance from the oval‐shaped chimney stent from the external pressure of the TAVR valve. The balloon then was used to assist the delivery of the guide extension. It was inflated at 10 atm (Figure 2), and during deflation, the guide extension was successfully delivered in the distal left main bifurcation (Figure 3). Then, the guidewire was used to cross the lesion on the circumflex artery. Typically, the lesion was predilated, and a drug‐eluting stent was deployed on the usual manner. After stent deployment, the guide extension was gently pulled back into the guide catheter, as the flow through the left main was obstructed and the patient got transiently hypotensive. Pulling the guide extension back allowed restoration of the normal flow through the left main and patient's hemodynamics immediately improved. Then, post‐dilation of the stent was performed with a non‐compliant balloon. At the end, balloon and wire were removed and multiple angiographic views showed TIMI III flow in both left anterior descending and circumflex arteries, without any changes in the anatomy of the left main ostium and all the hardware involved (Figure 4).

## 5 OUTCOME AND FOLLOW‐UP

The patient's angina disappeared, and 6 months after the procedure, patient continues to be asymptomatic. A cardiac stress MRI was performed 6 months post‐procedure that did not show recurrent ischemia in the lateral wall with preserved wall motion.

## 6 DISCUSSION

This case highlights the challenges performing percutaneous coronary intervention in the era of TAVR. Normally, with balloon‐expandable valves, accessing the coronaries is not considered to be a problem, because the stent frame is not high and usually stands lower than the coronary ostia. Rarely, as in this case, the low takeoff of the left main coronary artery in combination with the existing surgical bioprosthetic valve leaflets pose a threat, even with a balloon‐expandable valve; thus, a stent was deployed to protect the coronary flow. After the TAVR valve deployment, a residual high‐pressure gradient was measured. In order to reduce the gradient, a post‐dilation of the TAVR valve was performed, but the left main chimney stent was not protected with concurrent balloon inflation, resulting to oval‐shape deformation of the chimney stent. Performing percutaneous intervention through the left main chimney stent requires a thoughtful plan about the potential risks, including entrapment of the equipment (stent, wire, balloon), crossing through the side struts of the chimney stent and causing further deformation, leading to left main hypoperfusion and hemodynamic instability. Using a guide extension catheter protects the left main chimney stent by avoiding contact with all the necessary equipment for the percutaneous intervention. The balloon‐assisting technique was used to facilitate the passing of the guide extension through the left main chimney stent.

These cases will become more common as TAVR is gaining momentum. The case reported above according to our knowledge is the first percutaneous intervention through a left main chimney stent and can be a helpful resource while optimizing the methods of securing coronary artery access post‐TAVR.

### 6.1 Learning objectives

1. Importance of protecting the integrity of left main “chimney” stent while post‐dilating a TAVR valve
2. Improving safety while performing PCI through a left main “chimney” stent
3. Use of balloon‐assisted delivery of a guide extension catheter through a deformed stent

## AUTHOR CONTRIBUTIONS

The author planned and performed the procedure, wrote the manuscript, and made all necessary revisions.

## CONFLICT OF INTEREST

Nothing to disclose.

## CONSENT

Written informed consent was obtained from the patient to publish this report in accordance with the journal's patient consent policy.

## Data Availability

The data that support the findings of this case are available from the corresponding author upon reasonable request.
